# Role of long intergenic non-protein coding RNA 00152 in pancreatic cancer glycolysis *via* the manipulation of the microRNA-185-5p/Krüppel-like factor 7 axis

**DOI:** 10.7150/jca.63128

**Published:** 2021-08-28

**Authors:** Haifeng Li, Hao Shen, Peng Xie, Zheng Zhang, Lishan Wang, Yang Yang, Zeqian Yu, Zhangjun Cheng, Jiahua Zhou

**Affiliations:** 1 Department of Hepato-Pancreatico-Biliary Surgery, Zhongda Hospital, Medical School, Southeast University, 87 Dingjiaqiao Road, Nanjing, 210009, Jiangsu Province, China.; 2 Department of Hepato-Pancreatico-Biliary Surgery, Zhongda Hospital Southeast University, Nanjing, 210009, Jiangsu Province, China.

**Keywords:** Pancreatic cancer, Long intergenic non-protein coding RNA 00152, microRNA-185-5p, KLF7, Glycolysis, Competing endogenous RNA, Subcellular localization

## Abstract

The current study set out to investigate the role of long intergenic non-protein coding RNA (LINC) 00152 in pancreatic cancer (PC) cell glycolysis with the microRNA (miR)-185-5p/Krüppel-like factor 7 (KLF7) axis. Firstly, PC tissues and cells as well as the control ones were collected from 53 PC patients, and assessed for LINC00152 expression patterns. Besides, PC cells with the most differentially expressed LINC00152 were selected for further experiments. When LINC00152 was silenced or overexpressed, PC cell glucose consumption, lactic acid production, adenosine triphosphate and levels of glycolysis-associated enzymes were detected. In addition, the binding relation between LINC00152 and miR-185-5p as well as the target relation between miR-185-5p and KLF7 was clarified and validated. Additionally, xenograft transplantation was performed to confirm the *in vitro* experiments. It was found that LINC00152 was over-expressed in PC, and it predicted a poor prognosis. Besides, LINC00152 knockdown inhibited PC cell glycolysis. Moreover, LINC00152 could specifically targeted miR-185-5p. Meanwhile, LINC00152 exhaustion blocked PC cell glycolysis through the up-regulation of miR-185-5p. Lastly, LINC00152 inhibition targeted miR-185-5p to quench KLF7, therefore suppressing PC cell tumorigenesis and glycolysis. Collectively, our findings indicated that silencing LINC00152 restricted PC cell glycolysis *via* promoting miR-185-5p and reducing KLF7.

## Introduction

Pancreatic cancer (PC) represents a highly-fatal malignancy, accounting for skyrocketing rates of incidence and death across the world, in addition to unfavorable clinic outcomes and frustrating OS 1. Moreover, since PC cells metastasize to various other organs at a surprisingly rapid speed with no conspicuous symptoms at early stages, it remains particularly difficult to diagnose and control PC 2. Perilous causes such as diabetes, excessive smoking, overweight, poor dietary habit, heredity and lack of physical exercises have all been identified as the propellers of PC malignancy 3. Meanwhile, glycolysis is remarkably augmented in PC as a result of destructive cancer microenvironment and growing oncogenes, with the process facilitating carcinoma angiogenesis and epithelial-mesenchymal transition (EMT) to exasperate PC 4. Secondary injuries, such as parenchymal atrophy and pancreatic duct dilatation are regarded as the gold-standard for PC diagnosis, while molecular imaging and radiomics are also effective to detect and predict PC at quite early phase 5. In spite of this, PC remains a tremendous health burden and medical challenge in the form of unresectable tumors at advanced stages and widespread chemoresistance to a plethora of drugs 6. In this context, it is prudent to search for novel therapeutic strategies for PC.

Long non-coding RNAs (lncRNAs) are regarded as sanguine diagnostic and prognostic biomarkers for PC due to their ability to modulate PC occurrence and metastasis 7. Moreover, lncRNAs are capable of participating in cancer progression and glycolysis by virtue of adjusting cellular and genetic functions, modulating stem cell responses and mediating pathogen infection 8. One such lncRNA, LINC00152 is actively expressed in PC, and further encourages PC cell survival, invasion and viability 9. LINC00152 is similarly known to function as a tumor driver in numerous malignancies, such as lung cancer, kidney cancer, gastric cancer and gallbladder cancer by augmenting cancer cell aggressiveness, invasion and expansion 10. Furthermore, a prior study by *Tam et al*. illustrated lncRNAs are also capable of sponging microRNAs (miRs) to regulate mRNA, with the lncRNA/miR/mRNA axis being critical to homeostatic microenvironment and pathological changes of tumors 8. In addition, a recent study further highlighted miRs as constructive biomarkers in cancer identification and treatment as they regulate cell biological activities 11. More specifically, miR-185-5p has been shown to block invasion, dissemination and EMT, and enhance apoptosis in multiple cancers, such as hepatocellular carcinoma, breast cancer and prostate cancer 12-14. Furthermore, miR-185-5p is under-expressed in pancreatic ductal adenocarcinoma (PDAC) 15, which suggests that miR-185-5p can serve as a potential tumor suppressor in PC. Additionally, Krüppel-like factor 7 (KLF7) is known to be over-expressed in glioma, and further negatively-associated with patients' overall survival (OS) by augmenting cancer cellular progression and tumor formation 16. Also, KLF7 expression is activated in PDAC, and it leads to the induction of release of tumor-driver cytokines and PDAC cell expansion and development 17. Thence, the current study set out to investigate the crosstalk of LINC00152 in PC with the involvement of the downstream miR-185-5p/KLF7 axis.

## Materials and methods

### Ethics statement

The current study was approved and supervised by the Ethics committee of Zhongda Hospital. Animal experimentation protocols were also approved by the Institutional Animal Care and Use Committee of Zhongda Hospital and *International Convention on experimental Animal Ethics*. Signed informed consents were obtained from all participants prior to specimen collection. Extensive efforts were made to reduce the number and suffering of the experimental animals.

### Clinic tissue collection

A total of 53 PC patients undergoing excision at the Zhongda Hospital from October 2013 to October 2015, were enrolled in the current study for the collection of PC tissues and adjacent normal tissues 18, which were then deposited at -80 °C for further experimentation. All patients were histologically diagnosed as PC by 3 independent histopathologists. Subsequently, LINC00152 expression patterns were detected in the PC and adjacent normal tissues; with the median of LINC00152 expression serving as the threshold between highly- and lowly- expressed LINC00152. All patients (53 cases) received follow-up by telephone or return visits to observe the 5-year OS, which was defined as the period from the beginning of follow-up to the patient's death by any cause.

### Cell culture and transfection

Human normal pancreatic ductal cells hTERT-HPNE (HPNE) and PC cell lines BxPC-3, Capan-1, PANC-1 and CFPAC-1 (all from American Type Culture Collection, Manassas, Virginia, USA) 19 were procured and cultured in Dulbecco's modified Eagle's medium (Gibco Company, Grand Island, NY, USA) containing fetal bovine serum (Gibco) at 37℃ saturated humidity (Thermo Fisher Scientific Inc., Waltham, MA, USA) in a humidified incubator with 5% CO_2_. Upon reaching 90% density, the cells were detached with 0.25% trypsin (T1300, Science & Technology Co., Ltd., Beijing, China) and sub-cultured at a ratio of 1: 3, with the passage 2 cells used for further experimentation.

Cells were then transfected with miR-185-5p mimic, miR-185-5p inhibitor (IN), small interfere (si)-LINC00152, over-expression (oe)-LINC00152, oe-KLF7 and oe-negative control (NC) (all procured from Shanghai GenePharma Co., Ltd, Shanghai, China) in accordance with the instructions of Lipofectamine 2000 (Invitrogen Inc., Carlsbad, CA, USA) for a duration of 48 h for following use.

### Quantitative real-time polymerase chain reaction (qRT-PCR)

Total RNA was extracted from the aforementioned cells using RNeasy Mini kits (Qiagen, Valencia, CA, USA) and reverse-transcribed into cDNA with Reverse Transcription kits (RR047A, Takara Bio Inc., Kyoto, Japan). In addition, miR was detected using miR First Strand cDNA Synthesis (Tailing Reaction) kits (B532451-0020, Sangon Biotech Co., Ltd., Shanghai, China) and reverse-transcribed into cDNA. Next, qRT-PCR reaction was carried out with SYBR® Premix Ex Taq^TM^ II (Perfect RT) kits (DRR081, Takara) on a qRT-PCR instrument (ABI7500, ABI, Inc., Foster City, CA, USA). The amplification was divided into 2 processes; the first one was pre-denaturation at 95℃ for 30 s; the second one included 40 cycles of PCR reaction at 95℃ for 5 s and at 60 °C for 34 s, with 3 duplicate wells set for each sample. Primers were synthesized by Sangon (Table 1). Ct value of each well was recorded, with β-actin or U6 serving as the internal references. Relative expression of the samples was measured using the 2^-ΔCT^ method: ΔΔCt = (ΔCt experimental group-ΔCt control group) - (ΔCt=Ct target gene- Ct internal reference).

### Glucose consumption, lactic acid production and adenosine triphosphate (ATP) level assessment

The glucose oxidase method was adopted to determine glucose concentration. Cells at the logarithmic phase of growth were cultured in flasks with phenol red-free RPMI 1640 solution at density of 1.2×10^8^ cells/L for 48 h to measure glucose consumption. As per the instructions of the glucose kit (Sigma-Aldrich, St Louis, MO, USA), the 96-well plates were added with the corresponding regents and incubated at 37 °C for 15 min. Subsequently, the absorbance value of each well was measured at a wavelength of 505 nm using an automatic enzyme-linked immunosorbent assay system (ELx800, BioTek, USA). Glucose consumption = (glucose concentration in cell-free medium - glucose concentration in cell medium) × culture medium volume.

Additionally, lactic acid production was detected using the colorimetric method in accordance with the instructions of the lactic acid assay kits (A019-2, Nanjing JianCheng Bioengineering Institute, Nanjing, Jiangsu, China). The test medium was diluted to 5, 10 and 20 times, respectively, with the ideal value range of A value between 0.05-0.35. The results obtained from 20 times dilution were adopted. The absorbance value of standards and samples was measured at wavelength of 530 nm using an automatic enzyme-linked immunosorbent assay system.

Furthermore, ATP levels in the medium were measured with the help of ATP assay kits (A095-1, JianCheng). In brief, cell concentration was adjusted to 3 × 10^5^ cells/mL, and cells were seeded in 6-well plates (2 mL/well). The blank medium was set as the blank control for zero adjustment. ATP concentration (μmol/mg) = (absorbance value in the experimental group - absorbance value in the control group)/(absorbance value of standard - absorbance value in blank medium) × standard concentration (103 μmoL/L) × sample dilution ratio/total protein concentration (mg /L). Eventually, all values obtained were standardized as protein concentration 11.

### Western blot analysis

Cells and tissues were lysed with a radio-immunoprecipitation assay (RIPA) lysis buffer (Boster Biological Technology Co., Ltd, Wuhan, Hubei, China) consisting of protease inhibitor, and the protein concentration was determined using bicinchoninic acid protein quantification kits (Boster). The proteins were then transferred onto the polyvinylidene fluoride membranes following separation by 10% sodium dodecyl sulfate polyacrylamide gel electrophoresis. Next, the membranes were sealed with 5% bovine serum albumin for 2 h to block non-specific binding and cultivated with the following primary antibodies (all from Abcam Inc., Cambridge, MA, USA) hexokinase 2 (HK2) (dilution ratio of 1: 1000, ab209847), phosphofructokinase-2/fructose-2, 6-bisphosphatase 3 (PFKBF3) (dilution ratio of 1: 2000, ab181861), pyruvate dehydrogenase kinase 1 (PDK1) (dilution ratio of 1: 2000, ab207450), KLF7 (dilution ratio of 1: 2000, ab197690) and β-actin (dilution ratio of 1: 2000, ab8227) at 4 °C overnight. Afterwards, the membranes were incubated with horseradish peroxidase-labeled goat anti-rabbit immunoglobulin G (IgG) antibody (dilution ratio of 1: 2000, ab205718, Abcam) for 1 h. Subsequently, the membranes were developed with enhanced chemiluminescence working fluid (EMD, Merck Millipore Corp., Billerica, MA, USA). Lastly, the Image Pro Plus 6.0 software (Media Cybernetics, San Diego, CA, USA) was employed to quantify the gray value of band in different groups, with β-actin as the internal reference. All experiments were performed thrice to obtain the mean value.

### Fluorescence *in situ* hybridization (FISH) on LINC00152 subcellular localization

LINC00152 subcellular localization was identified by means of a FISH assay. Following the instructions of the Ribo^TM^ lncRNA FISH probe Mix (Red, Guangzhou RiboBio Co., Ltd., Guangzhou, Guangdong China), coverslips were placed on 6-well plates, with cells seeded in the plates for 1 h-incubation to ensure the cell confluence approached 80%. After removing the coverslips, the cells were subjected to a phosphate buffer saline (PBS) rinse, fixation with 1 mL 4% paraformaldehyde, treatment by protease K ((2 μg/mL), glycine and acetylation reagent, and then cultured in 250 μL prehybridization solution at 42 °C for 1 h. Subsequently, upon discarding the prehybridization solution, the coverslips were cultivated with 250 μL hybridization solution containing probe (300 ng/mL) at 42℃ overnight. Afterwards, the coverslips were rinsed with PBS containing 0.05% (v/v) Tween-20 (PBST) three times, treated by PBST-diluted 4', 6-diamidino-2-phenylindole (1: 800) staining solution to satin nucleus and seeded in 24-well plates for 5 min-staining. After another 3 PBST rinses (3 min/time) and sealing with anti-fluorescent exhaustion reagent, five different visual fields were selected for the observation and photographing (400×) under a fluorescence microscope (Olympus Optical Co., Ltd, Tokyo, Japan) 20.

### Dual-luciferase reporter gene assay

To further verify the direct target relation between LINC00152 and miR-185-5p, and between miR-185-5p and KLF7, synthesized LINC00152-wild type (WT), KLF7-WT, LINC00152-mutant type (MUT) and KLF7-MUT were constructed on pMIR-reporter plasmids (Beijing Huayueyang Biotechnology Inc, Beijing, China). Next, these above-mentioned luciferase reporter plasmids and miR-185-5p mimic were co-transfected into HEK293T cells (Shanghai Beinuo Biotechnology Co., Ltd., Shanghai, China) for 48 h. Afterwards, the cells were collected and lysed using luciferase assay kits (K801-200, BioVision Inc., Mountain View, CA, USA).

### RNA immunoprecipitation (RIP)

RIP kits (Merck Millipore Corp., Billerica, MA, USA) were adopted to certify the target relation between LINC00152 and miR-185-5p, and between miR-185-5p and KLF7. Cells in all groups were rinsed with pre-cooled PBS to discard the supernatant. Next, the cells were lysed with same amounts of iced RIPA lysis buffer, and then centrifuged at 4℃ for 10 min to discard supernatant. A portion of the cell extraction solution was stored as the Input and the remaining portion was co-precipitated *via* culturing with antibodies. RNA was separated from samples after being detached with proteinase K and used for subsequent qRT-PCR on the expression patterns of LINC00152, miR-185-5p and KLF7. The following antibodies were adopted for RIP: rabbit anti-Ago2 (dilution ratio of 1: 100, ab32381, Abcam) (mixed for 30 min) and rabbit anti-IgG (dilution ratio of 1:100, ab109489) (as the NC). All experiments were performed thrice to obtain the mean value.

### Xenografts tumors in nude mice

A total of 36 female nude mice (aged 5-6-week-old) (Shanghai SLAC Laboratory Animal Co., Ltd., Shanghai, China) were housed in 25-27 °C environment with constant humidity. All the lentivirus (LV)-short hairpin (sh)-NC, LV-sh-LINC00152, LV-oe-NC and LV-oe-KLF7 were purchased from Shanghai GenePharma Co, Ltd. (Shanghai, China). Upon reaching 80-90% cell growth rate, the PANC-1 cells were challenged by the above LV with the existence of 10 mg/mL polybrene (MOI=10, 21. PANC-1 cells with stable low-expression of LINC00152 or stable over-expression of KLF7 were obtained by Zeocin or Neomycin resistance screening. Afterwards, the cells were detached, centrifuged, rinsed with PBS thrice and resuspended for cell number calculation. Upon adjusting the cell density to 1×10^7^ cells/mL, 20 μL cell suspension was injected into nude mice *via* the axilla (n=12). Tumor size, volume and growth curve were assessed every week, and tumor volume was measured as V=(a×b^2^)/2, with a representing length, and b representing width. Mice were euthanatized using administration of pentobarbital sodium (100 mg/kg) after 4 weeks. Afterwards, the tumors were removed and weighed. Six randomly selected mice were used for qRT-PCR and Western blot analysis, and the remaining 6 mice were used for paraffin-embedding and section slicing.

### Immunohistochemistry

Tumor tissues were fixed in 4% paraformaldehyde solution, paraffin-embedded and sectioned (5 μm). After dewaxing and hydration, the slides were immersed in 10 mM citrate buffer (pH = 7.5) and antigen-repair was performed with a microwave. The endogenous peroxidase activity was blocked with the addition of 3% hydrogen peroxide. Next, the sections were incubated with diluted anti-ki67 antibody (dilution ratio of 1:200, ab15580, Abcam) at 4 °C overnight in a humidified chamber, and then incubated with secondary antibody IgG (dilution ratio of 1:200, ab150077, Abcam) at room temperature for 1 hour. The sections were then stained with hematoxylin after DAB staining. Lastly, the sections were sealed and observed under a microscope.

### Statistical analysis

Statistical analyses were performed using the SPSS 21.0 software (IBM Corp. Armonk, NY, USA). Measurement data conformed to normal distribution and were shown in mean ± standard deviation. The *t-test* was adopted to analyze comparisons between two groups, and one-way or two-way analysis of variance (ANOVA) to compare different groups, and Tukey's post-hoc multiple comparisons test to perform pairwise comparisons after ANOVA. The Kaplan-Meier method was employed to analyze the survival curve of patients, and log-rank test was used to analyze survival difference of patients. A value of *p* < 0.05 was regarded statistically significant.

## Results

### LINC00152 is over-expressed in PC and predicts poor prognoses

**Figure 1:**
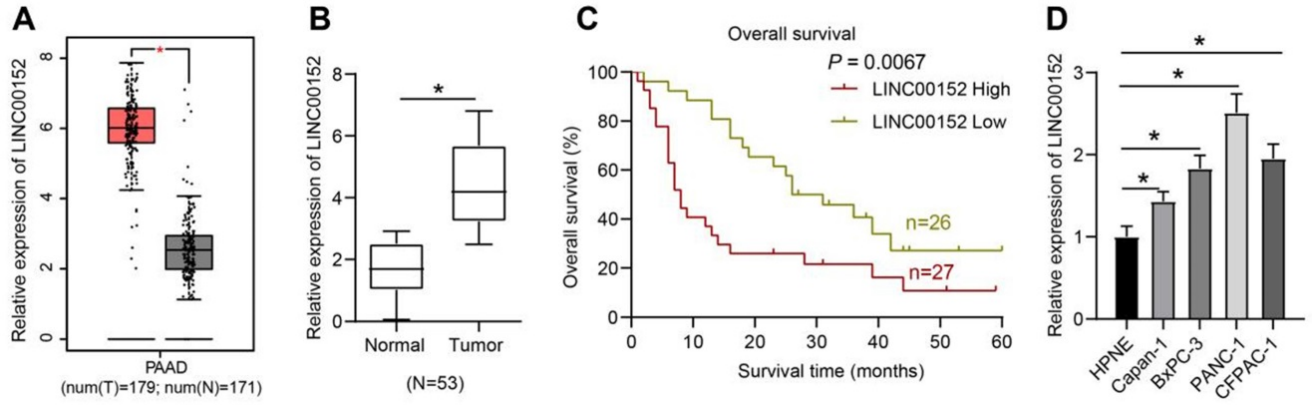
** LINC00152 is over-expressed in PC, and it predicts poor prognosis. A,** LINC00152 over-expression in PC predicted by GEPIA website. **B,** LINC00152 expression measured by qRT-PCR, the paired t-test was employed to test statistical significance; n=53. The n specifies the number of tissue case. **C,** The relation between LINC00152 expression and PC patients' prognosis analyzed by Kaplan-Meier and log-rank test; n=53. The n specifies the number of tissue case. **D,** LINC00152 expression in human normal pancreatic ductal cells and PC cells assessed through qRT-PCR; n=3. The n specifies the replicate. The results were exhibited as mean ± standard deviation. One-way ANOVA was used to analyze data. Tukey's multiple comparisons test was applied for post hoc test. * *p*<0.05.

LINC00152 was predicted to be over-expressed in PC as revealed by the GEPIA website (http://gepia.cancer-pku.cn/detail.php?gene=LINC00152) (Fig. 1A). Firstly, we collected 53 pairs of PC tissues and adjacent normal tissues and detected the expression patterns of LINC00152 using qRT-PCR. It was found that compared with the adjacent normal tissues, PC tissues presented with high LINC00152 expressions (*p*<0.05) (Fig. 1B). As the relation between LINC00152 expression and PC patients' prognosis was subsequently determined, the median of LINC00152 expression was identified as the threshold between strongly- and poorly- expressed LINC00152, which revealed that 5-year OS of patients with strong LINC00152 expression was less compared to those with poor LINC00152 expressions (*p*<0.05) (Fig. 1C). In addition, LINC00152 expression patterns in HPNE cells as well as BxPC-3, Capan-1, PANC-1 and CFPAC-1 cells were verified through qRT-PCR, which demonstrated that compared with HPNE, PC cells presented with highly-expressed LINC00152 (*p*<0.05) (Fig. 1D). Thus, among the PC cell lines, PANC-1 cells presenting with the highest LINC00152 expression and Capan-1 with the lowest LINC00152 expression were selected for further experimentation.

### LINC00152 knockdown inhibits PC cell glycolysis

**Figure 2:**
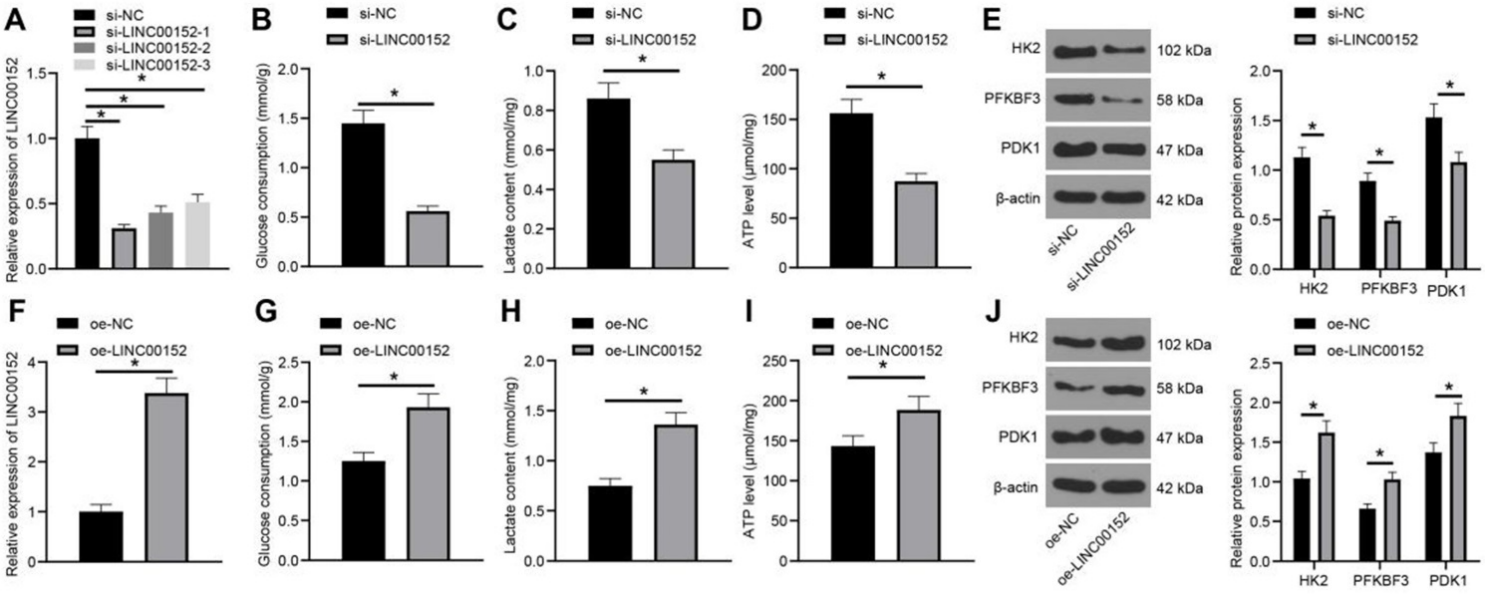
** LINC00152 knockdown inhibits PC cell glycolysis. A,** LINC00152 expression in PANC-1 cells with si-LINC00152 detected qRT-PCR. **B, C and D,** Glucose consumption (B), lactic acid production (C) and ATP level (D) in PANC-1 cells with si-LINC00152. **E,** Levels of glycolysis-related enzymes in PANC-1 cells with si-LINC00152 assessed by western blot analysis. **F,** LINC00152 expression in Capan-1 cells with oe-LINC00152 detected qRT-PCR. **G, H and I,** Glucose consumption (G), lactic acid production (H) and ATP level (I) in Capan-1 cells with oe-LINC00152. **J,** Levels of glycolysis-correlated enzymes in PANC-1 cells with si-LINC00152 assessed by western blot analysis. In panels A-J, n=3. The n specifies the replicate. The results were exhibited as mean ± standard deviation. One-way ANOVA was used to analyze data in panel A. Two-way ANOVA was used to analyze data in panels E and J. Tukey's multiple comparisons test was applied for post hoc test. Independent t-test was used to analyze data in B, C, D, F and I * *p*<0.05.

To further examine the function of LINC00152 in PC cell glycolysis progression, LINC00152 was down-regulated in PANC-1 cells and detected *via* qRT-PCR, which revealed that the si-LINC00152-1 group, si-LINC00152-2 group and si-LINC00152-3 group all exhibited reduced LINC00152 expressions compared to the si-NC group (*p*<0.05) (Fig. 2A). Since si-LINC00152-1 (si-LINC00152) showed the most pronounced silencing efficiency, it was selected for subsequent experiments. Glucose consumption, lactic acid production and ATP levels in PC cells were then measured, which demonstrated that the si-LINC00152 group exhibited declined glucose consumption, lactic acid production and ATP levels compared with the si-NC group (*p*<0.05) (Fig. 2B-D). In addition, levels of glycolysis-related enzymes (HK2, PFKBF3 and PDK1) were examined *via* Western blot analysis, and it was found that levels of these enzymes were all lower in the si-LINC00152 group than those in the si-NC group (*p*<0.05) (Fig. 2E).

Similarly, LINC00152 was over-expressed in Capan-1 cells and assessed, and qRT-PCR demonstrated that LINC00152 was up-regulated in the oe-LINC00152 group relative to the oe-NC group (*p*<0.05) (Fig. 2F). Glucose consumption, lactic acid production and ATP level were also examined, which revealed that the oe-LINC00152 group showed elevated glucose consumption, lactic acid production and ATP levels compared with the oe-NC group (*p*<0.05) (Fig. 2G-I). Subsequently, levels of HK2, PFKBF3 and PDK1 were assessed by Western blot analysis, and it was discovered that levels of HK2, PFKBF3 and PDK1 were all promoted in the si-LINC00152 group relative to those in the si-NC group (*p*<0.05) (Fig. 2J). Altogether, these findings indicated that LINC00152 knockdown could suppress PC cell glycolysis; while LINC00152 over-expression brought about the opposite results.

### LINC00152 sponges miR-185-5p, whose overexpression reduces PC cell glycolysis

**Figure 3:**
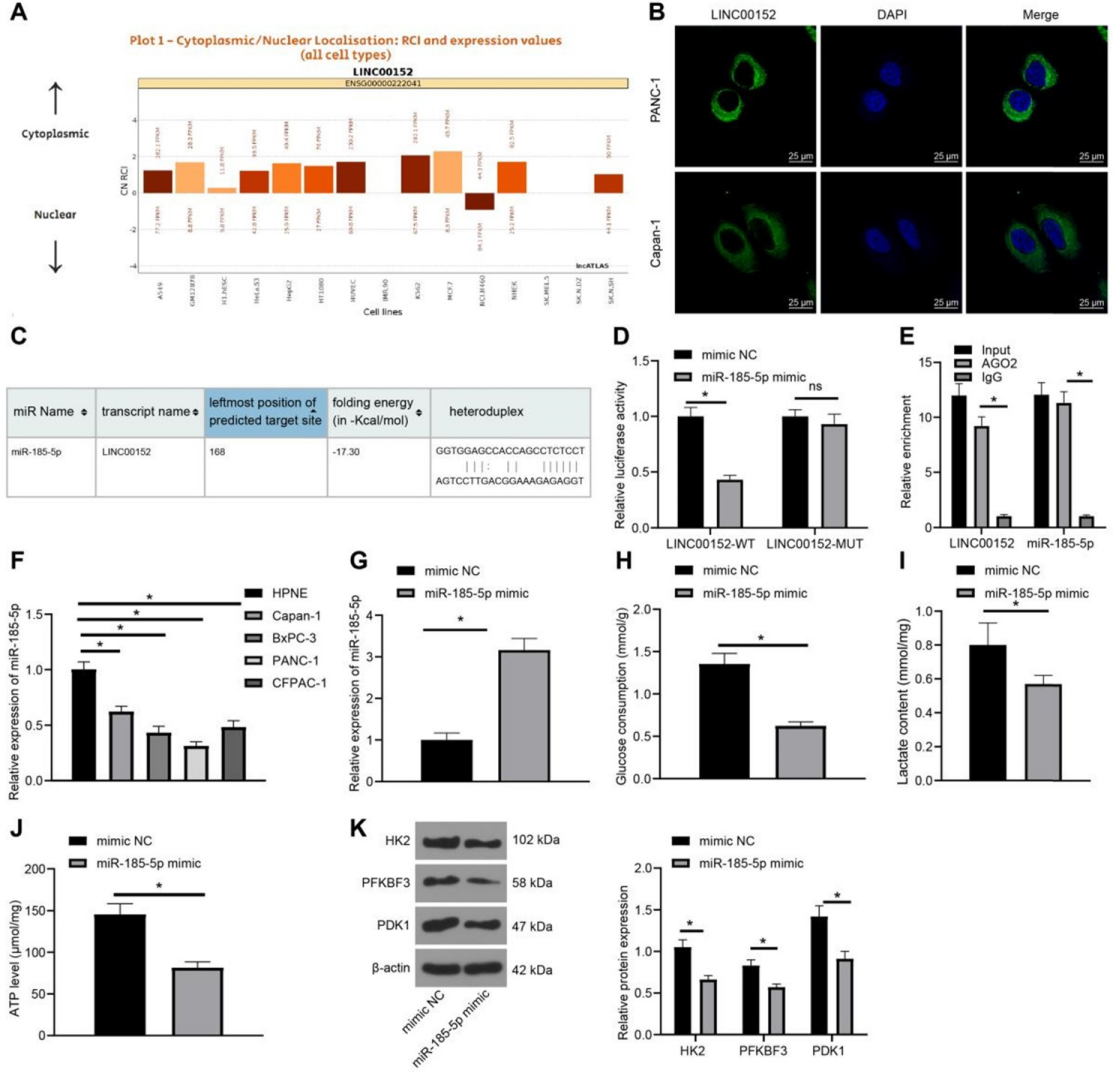
** LINC00152 sponges miR-185-5p, whose overexpression reduces PC cell glycolysis. A,** LINC00152 principal localization at cytoplasm predicted by website (http://lncatlas.crg.eu/). **B,** LINC00152 subcellular localization in PANC-1 and Capan-1 certified by FISH, 400×, scale bar: 25 µm. **C,** the binding relation between LINC00152 and miR-185-5p predicted by RNA22 website. **D,** The target relation of LINC00152 and miR-185-5p confirmed by dual-luciferase reporter gene assay. **E,** The target relation of LINC00152 and miR-185-5p detected by RIP. **F,** miR-185-5p expression in human normal pancreatic ductal cells and PC cells determined by qRT-PCR. **G,** miR-185-5p expression in PANC-1 transfected with mimic NC or miR-185-5p mimic examined through qRT-PCR. **H, I and J,** Glucose consumption (H), lactic acid production (I) and ATP level (J) in PC cells with mimic NC and miR-185-5p mimic. **K,** Levels of glycolysis-related enzymes detected by western blot analysis. In panels B/D-K, n=3. The n specifies the replicate. The results were exhibited as mean ± standard deviation. Two-way ANOVA was used to analyze the data in panels D, E, F and K. Tukey's multiple comparisons test was applied for the post hoc test. Independent t-test was used to analyze data in G, H, I and J. * *p*<0.05.

In order to elucidate the downstream mechanism of LINC00152, an online prediction database (http://lncatlas.crg.eu/) was retrieved, which revealed that LINC00152 was primarily localized in the cytoplasm (Fig. 3A). Subsequent FISH assay verified that LINC00152 subcellular localization in PANC-1 and Capan-1 cells was also located in the cytoplasm (Fig. 3B), suggesting that LINC00152 could function *via* the competing endogenous RNA (ceRNA) network in PC. The RNA22 website (https://cm.jefferson.edu/rna22/Interactive/RNA22Controller) was further employed to predict the downstream miR that could bind to LINC00152, which indicated that LINC00152 could bind to miR-185-5p (Fig. 3C). In addition, it was found that compared with the mimic NC group, the miR-185-5p mimic group exhibited decreased LINC00152-WT luciferase activity (*p*<0.05) (Fig. 3D), but LINC00152-MUT luciferase activity was unaffected. Moreover, RIP assay on PANC-1 cells revealed that AGO2-combined LINC00152 and miR-185-5p expressions were up-regulated compared with IgG (*p*<0.05) (Fig. 3E). Furthermore, miR-185-5p expression patterns in HPNE and PC cell lines were detected using qRT-PCR, and miR-185-5p expressions were found to be down-regulated in PC cell lines relative to HPNE cells(*p*<0.05) (Fig. 3F). In that sense, these findings indicated that LINC00152 and miR-185-5p were specifically bound in PC cells.

Additionally, mimic NC or miR-185-5p mimic were transfected into PANC-1 cells, and subsequent results of qRT-PCR demonstrated that miR-185-5p was over-expressed in the miR-185-5p mimic group (*p*<0.05) (Fig. 3G). Meanwhile, detection of glucose consumption, lactic acid production and ATP levels in PC cells revealed that compared with the mimic NC group, the miR-185-5p mimic group showed discouraged glucose consumption, lactic acid production and ATP levels (*p*<0.05) (Fig. 3H-J). Likewise, levels of HK2, PFKBF3 and PDK1 were determined by Western blot analysis, and it was found that levels of HK2, PFKBF3 and PDK1 were all quenched in the miR-185-5p mimic group compared with those in the mimic NC group (*p*<0.05) (Fig. 3K). Altogether, these findings demonstrated that over-expression of miR-185-5p inhibited PC cell glycolysis.

### Silencing LINC00152 suppresses PC cell glycolysis *via* encouraging miR-185-5p expression

**Figure 4:**
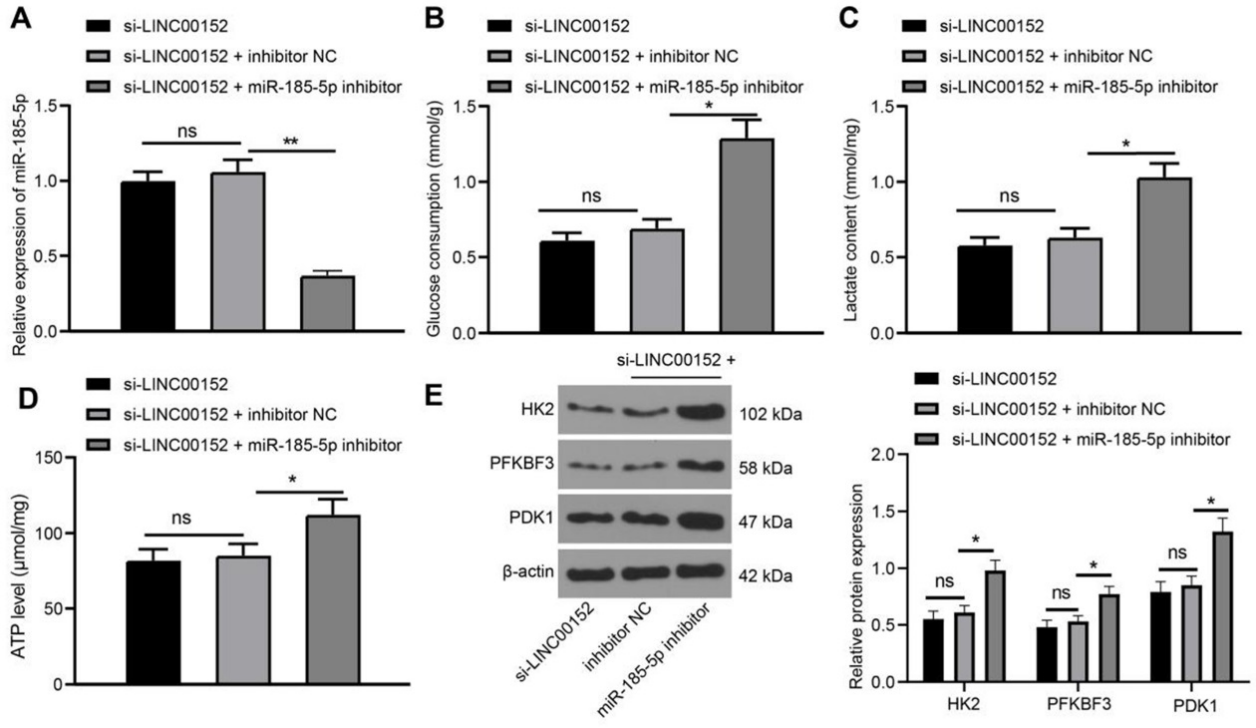
** Silencing of LINC00152 suppresses PC cell glycolysis via encouraging miR-185-5p expression. A,** miR-185-5p expression verified by qRT-PCR. **B, C and D,** Glucose consumption (B), lactic acid production (C) and ATP level (D) in PC cells. **E,** Levels of glycolysis-related enzymes determined by western blot analysis. In panels A-E, n=3. The n specifies the replicate. The results were exhibited as mean ± standard deviation. One-way ANOVA was used to analyze the data in panels A, B, C and D. Two-way ANOVA was used to analyze the data in panel E. Tukey's multiple comparisons test was applied for post hoc test. * *p*<0.05.

Functional rescue assay was further performed as LINC00152 and miR-185-5p were simultaneously silenced in PANC-1 cells to assess miR-185-5p expression *via* qRT-PCR, which revealed that miR-185-5p was under-expressed in the si-LINC00152+miR-185-5p-IN group as compared with those in the si-LINC00152+IN-NC group (*p*<0.05) (Fig. 4A); whereas, miR-185-5p expression in the si-LINC00152 group and the si-LINC00152+miR-185-5p-IN group showed no statistical differences. Meanwhile, assessment of glucose consumption, lactic acid production and ATP levels demonstrated that the si-LINC00152+miR-185-5p-IN group exhibited elevated glucose consumption, lactic acid production and ATP levels compared to the si-LINC00152+IN-NC group (*p*<0.05) (Fig. 4B-D); while the si-LINC00152 group and si-LINC00152+IN-NC group exhibited no statistical difference. Moreover, levels of HK2, PFKBF3 and PDK1 were assessed by Western blot analysis, which indicated that the si-LINC00152+miR-185-5p-IN group exhibited higher levels of HK2, PFKBF3 and PDK1 relative to the si-LINC00152+IN-NC group (*p*<0.05) (Fig. 4E), whereas the si-LINC00152 group and si-LINC00152+IN-NC group didn't exhibit any statistical difference. Overall, these findings suggested that silencing LINC00152 inhibited PC cell glycolysis by over-expressing miR-185-5p.

### Silencing LINC00152 down-regulates KLF7 expression *via* the upregulation of miR-185-5p

**Figure 5:**
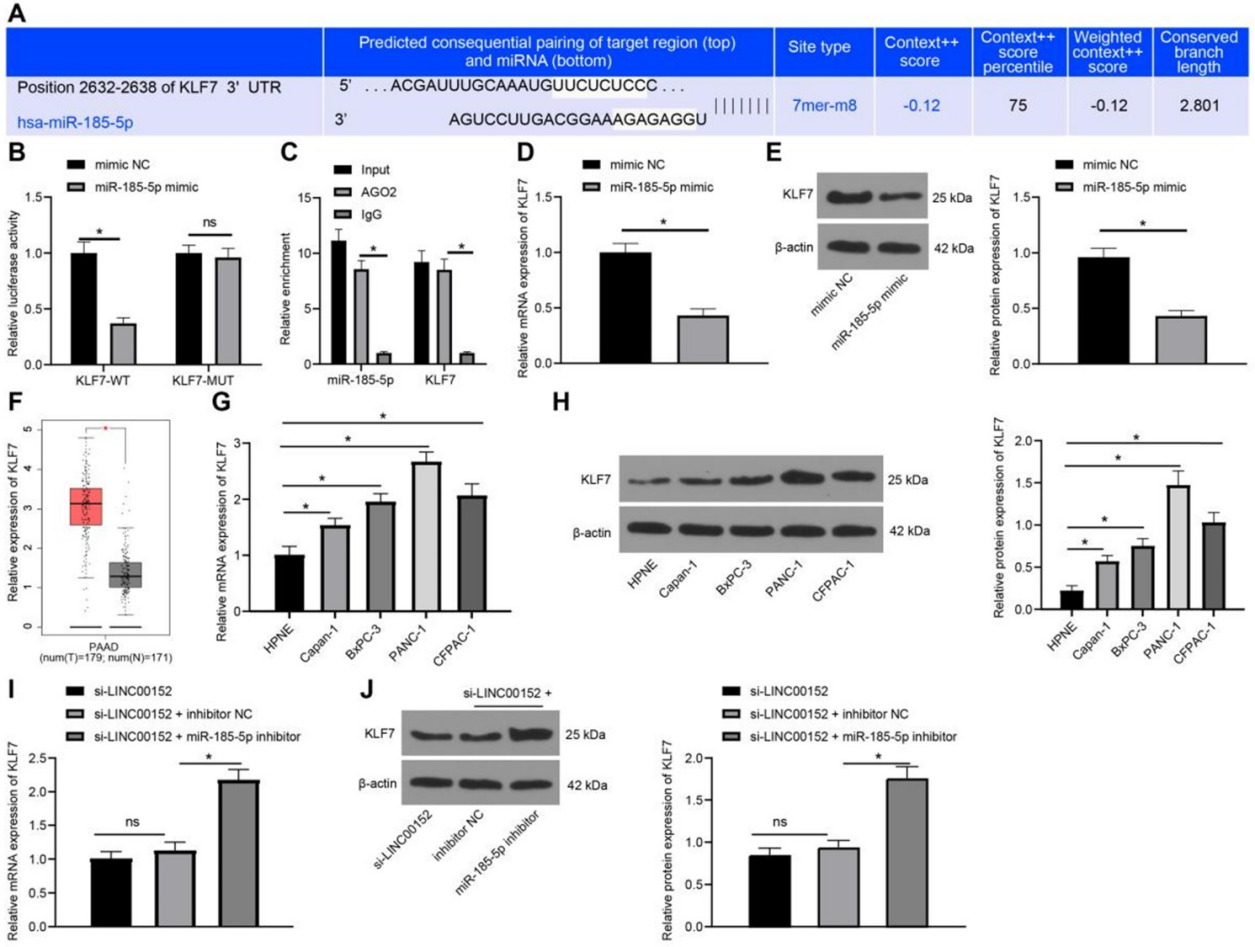
** Silencing of LINC00152 downregulates KLF7 expression via the upregulation of miR-185-5p. A,** miR-185-5p binding to KLF7 at 3'UTR predicted by TargetScan website. **B,** miR-185-5p binding to KLF7 at 3'UTR verified via dual-luciferase reporter gene assay. **C,** miR-185-5p binding to KLF7 detected via RIP. **D,** KLF7 expression in PANC-1 cells transfected with mimic NC and miR-185-5p mimic assessed by qRT-PCR. **E,** KLF7 expression measured by western blot analysis. **F,** Highly expressed KLF7 in PC predicted by GEPIA website. **G, H, I and J,** KLF7 expression in PC cells examined via qRT-PCR (G and I) and western blot analysis (H and J). In panels B-E and G-J, n=3. The n specifies the replicate. The results were exhibited as mean ± standard deviation. The independent t-test was used to analyze the data in panels D and E. One-way ANOVA was used to analyze the data in panels G, H, I and J. Two-way ANOVA was used to analyze the data in panels B and C. Tukey's multiple comparisons test was applied for the post hoc test. * *p*<0.05.

The Targetscan website (http://www.targetscan.org/cgi-bin/targetscan/vert_71/) was retrieved to explore the effect of miR-185-5p in PC, which predicted that miR-185-5p could bind to KLF7 at 3'UTR (Fig. 5A). Meanwhile, the results of dual-luciferase reporter gene assay revealed that luciferase activity of KLF7-WT was degraded in the miR-185-5p mimic compared with that in the mimic NC group (*p*<0.05) (Fig. 5B), while the luciferase activity of KLF7- MUT was unchanged, validating that miR-185-5p bound to KLF7 at 3'UTR. In addition, the binding relation between miR-185-5p and KLF7 was verified with a RIP assay (*p*<0.05) (Fig. 5C). Furthermore, KLF7 expression patterns in PANC-1 cells transfected with mimic NC and miR-185-5p mimic were also detected *via* qRT-PCR and Western blot analysis, which revealed that the miR-185-5p mimic group exhibited reduced KLF7 expressions compared to the mimic NC group (*p*<0.05) (Fig. 5D-E).

Additionally, online analyses with the GEPIA website (http://gepia.cancer-pku.cn/detail.php?gene=KLF7) indicated that KLF7 was over-expressed in PC (p<0.05) (Fig. 5F). Subsequently, KLF7 expression patterns in HPNE cells as well as BxPC-3, Capan-1, PANC-1 and CFPAC-1 cells were measured through qRT-PCR and Western blot analysis, which revealed that PC cells presented with highly-expressed KLF7 compared to HPNE cells (*p*<0.05) (Fig. 5G-H). In addition, when LINC00152 and miR-185-5p were both depleted in PANC-1 cells, KLF7 expression patterns were detected again with qRT-PCR and Western blot analysis, and it was found that KLF7 expression was increased in the si-LINC00152+miR-185-5p-IN group compared with the si-LINC00152+IN-NC group (*p*<0.05) (Fig. 5I-J); while the si-LINC00152 group and the si-LINC00152+IN-NC group exhibited no statistical difference. Collectively, these findings suggested that KLF7 was highly-expressed in PC cells, and it was down-regulated by silencing LINC00152 *via* miR-185-5p up-regulation.

### Silencing LINC00152 reduces PC cell glycolysis by inhibiting KLF7 expression

**Figure 6:**
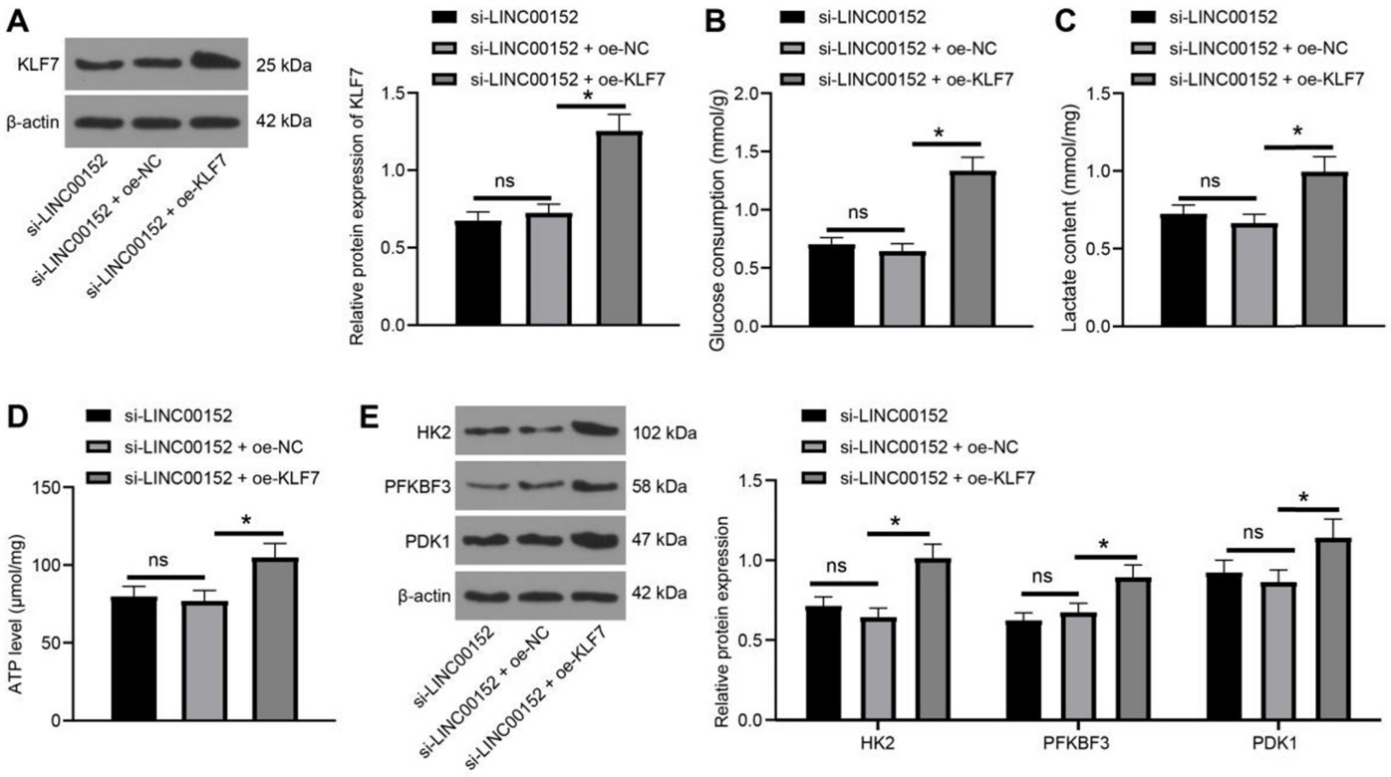
** Silencing of LINC00152 reduces PC cell glycolysis by inhibiting KLF7 expression. A,** KLF7 expression measured by western blot analysis. **B, C and D,** glucose consumption (B), lactic acid production (C) and ATP level (D) in PC cells. **E,** Levels of glycolysis-related enzymes determined by western blot analysis. In panels A-E, n=3. The n specifies the replicate. The results were exhibited as mean ± standard deviation. One-way ANOVA was used to analyze the data in panels A, B, C and D. Two-way ANOVA was used to analyze the data in panel E. Tukey's multiple comparisons test was applied for the post hoc test. * *p*<0.05.

To further validate the regulation of glycolysis of LINC00152 was facilitated by KLF7, LINC00152 was silenced in PANC-1 cells and KLF7 was over-expressed. Subsequent KLF7 expression patterns were analyzed through Western blot analysis, which revealed that KLF7 expression levels were higher in the si-LINC00152+oe-KLF7 group than that in the si-LINC00152+oe-NC group (*p*<0.05) (Fig. 6A), whereas the si-LINC00152 group and si-LINC00152+oe-NC group exhibited no statistical differences. Moreover, glucose consumption, lactic acid production and ATP levels were determined, and it was found that compared with the si-LINC00152+oe-NC group, the si-LINC00152+oe-KLF7 group presented with increased glucose consumption, lactic acid production and ATP levels (*p*<0.05) (Fig. 6B-D), while the si-LINC00152 group and si-LINC00152+oe-NC group didn't show any statistical differences. Meanwhile, levels of HK2, PFKBF3 and PDK1 were examined with Western blot analysis, which demonstrated that levels of these enzymes were all increased in the si-LINC00152+oe-KLF7 group compared with those in the si-LINC00152+oe-NC group (*p*<0.05) (Fig. 6E), while there were no statistical differences between the si-LINC00152 group and the si-LINC00152+oe-NC group, indicating that silencing LINC00152 inhibited KLF7 expression to restrict PC cell glycolysis.

### Silencing LINC00152 limits PC tumorigenesis and glycolysis *via* the miR-185-5p/KLF7 axis

**Figure 7:**
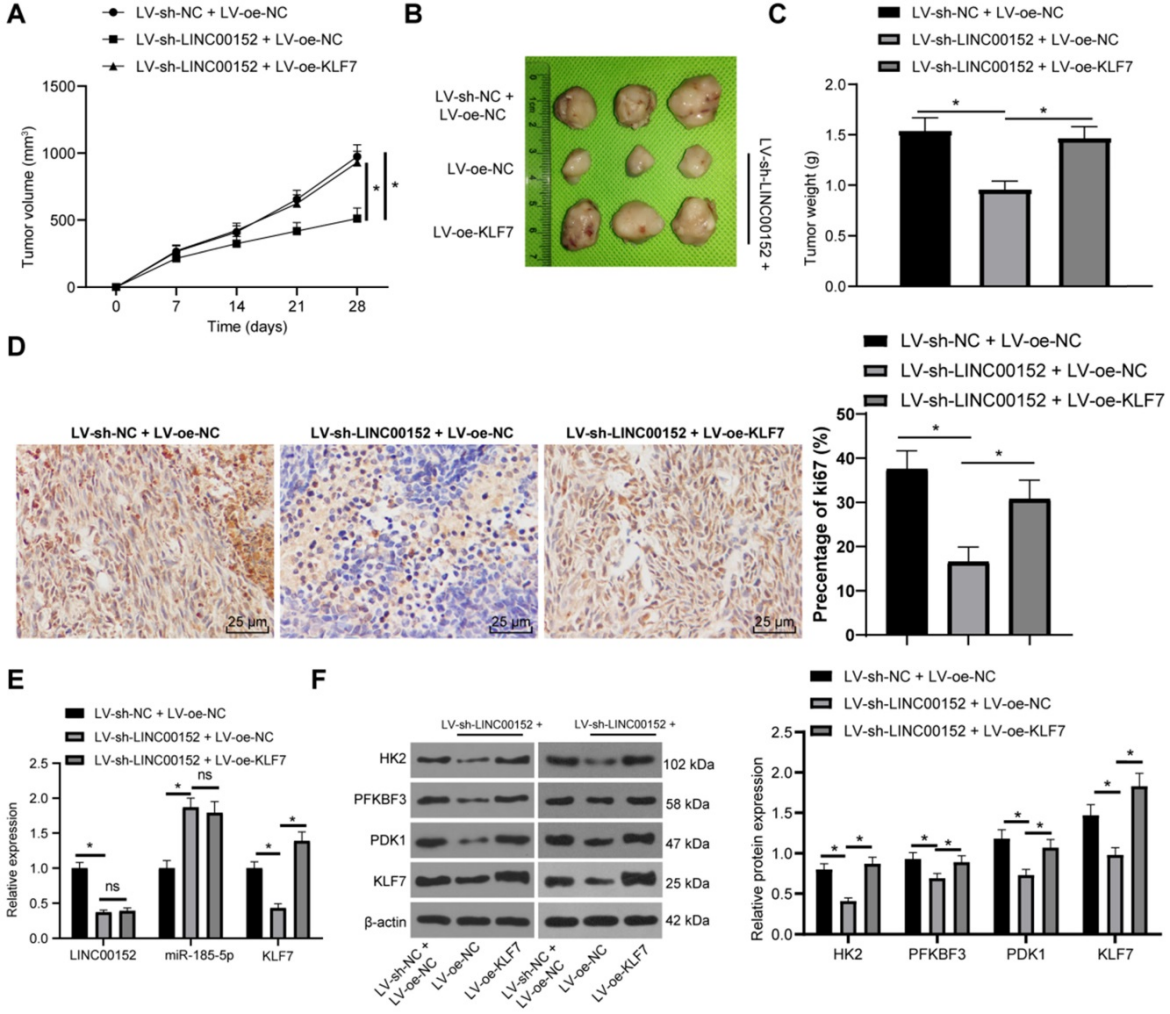
** LINC00152 knockout discourages PC cell tumorigenesis and glycolysis by quenching KLF7 expression.** The lentivirus interference vector (LV-sh-LINC00152) and lentivirus overexpression vector KLF7 (LV-oe-KLF7) as well as their controls were infected with PANC-1 cells. Then the stably-expressed cells were screened for subcutaneous tumor formation in nude mice. **A,** Tumor growth; n=12, the n specifies the mouse number. **B,** Representative images of tumor. **C,** Tumor weight; n=12, the n specifies the mouse number. **D,** Positive rate of Ki67 in tumor tissue detected by Immunohistochemistry. **E,** Expression of LINC00152, miR-185-5p and KLF7 measured by qRT-PCR. **F,** Glycolysis-related enzyme levels and KLF7 assessed via western blot analysis; the image on the left is a representative image of Western blot in tumor tissue. In panels D-E, n=6, the n specifies the mouse number; the results were exhibited as mean ± standard deviation. One-way ANOVA was used to analyze the data in panel C. Two-way ANOVA was used to analyze the data in panels A, D and E. Tukey's multiple comparisons test was applied for the post hoc test. * *p*<0.05.

Lastly, the role of LINC00152 in PC cell glycolysis *via* the miR-185-5p/KLF7 axis was confirmed *in vivo*. After the establishment of xenografts tumors, tumor growth and weights were both found to be lower in the LV-sh-LINC00152+LV-oe-NC than those in the LV-sh-NC+LV-oe-NC group, but higher in the LV-sh-LINC00152+LV-oe-KLF7 group than those in the LV-sh-NC+LV-oe-NC group (Fig. 7A-C). Compared with the LV-sh-NC + LV-oe-NC group, the positive rate of Ki67 in the LC-sh-LINC00152 + LV-oe-NC group was significantly lowered. Meanwhile, compared with the LV-sh-LINC00152 + LV-oe-NC group, the positive rate of Ki67 in the LV-sh-LINC00152 + LV-oe-KLF7 group was significantly elevated (Fig. 7D). In addition, the expression patterns of LINC00152, miR-185-5p and KLF7 were detected by qRT-PCR, which revealed that the LV-sh-LINC00152+LV-oe-NC group presented with lower LINC00152 and KLF7 expressions compared to the LV-sh-NC+LV-oe-NC group; while compared with the LV-sh-LINC00152+LV-oe-NC group, the LV-sh-LINC00152+LV-oe-KLF7 group showed promoted KLF7 expression levels (Fig. 7E). Moreover, HK2, PFKBF3, PDK1 and KLF7 levels were verified through Western blot analysis, and the levels of these enzymes were all found to be diminished in the LV-sh-LINC00152+LV-oe-NC group compared with those in the LV-sh-NC+LV-oe-NC group; on the other hand, compared with the LV-sh-LINC00152+LV-oe-NC group, the LV-sh-LINC00152+LV-oe-KLF7 expressed increased the levels of the above enzymes (Fig. 7F). Collectively, these findings indicated that LINC00152 knockout discouraged PC tumorigenesis and glycolysis by quenching KLF7 expression.

## Discussion

**Figure 8:**
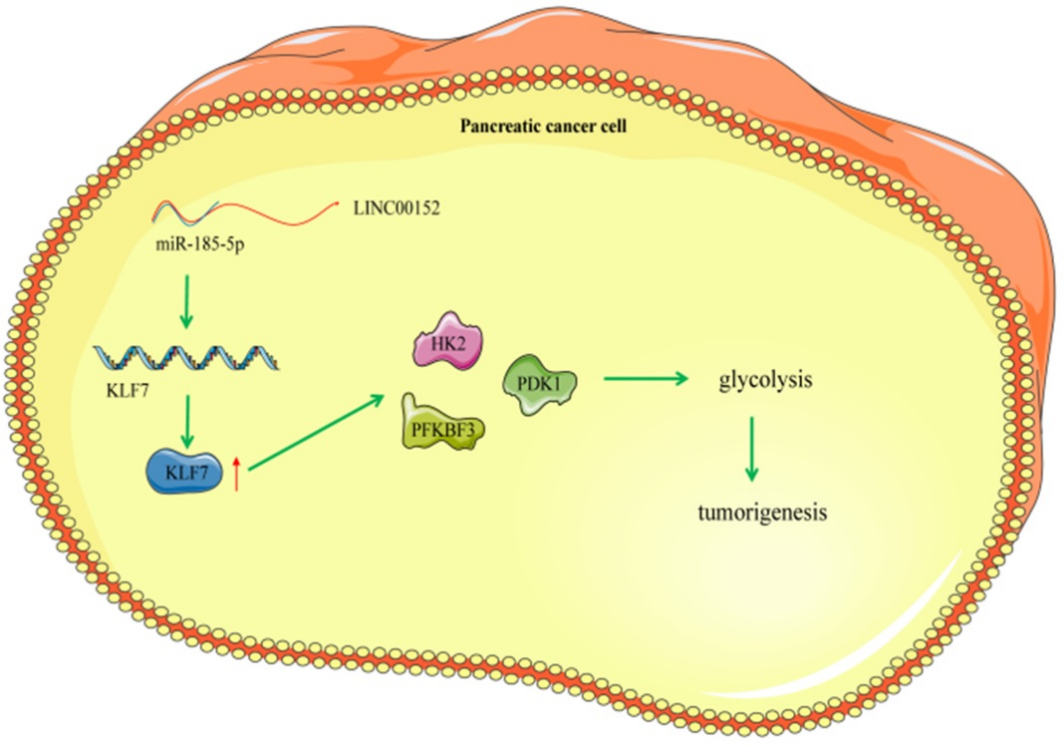
** Mechanism diagram.** LINC00152 promotes the expression of KLF7 through the competitive binding to miR-185-5p in pancreatic cancer cells, thereby promoting the glycolysis process of pancreatic cancer cells and promoting the occurrence of pancreatic tumor.

PC represents a fairly common life-threatening neoplasm, characterized by strong aggressiveness and high rates of morbidity and mortality affecting the male and elderly populations 2. Meanwhile, lncRNAs, a family of RNA molecules that possess the ability to modulate protein activities, are known to exert crucial effects on carcinoma occurrence, metabolism and glycolysis 22. One particular lncRNA, namely LINC00152 was previously highlighted to be differential expressed in pancreatic ductal adenocarcinoma, and further implicated in PDAC identification, detection and regulation 23. Therefore, it would be plausible to suggest that LINC00152 could function as a significant regulator in PC progression. However, only a handful of studies have explored the role of LINC00152 in PC, let alone the crosstalk of LINC00152 and other downstream axis in the biological behaviors of PC. Consequently, the current study set out to elucidate the potential role of LINC00152 in PC, and our obtained findings indicated that silencing LINC00152 alleviated PC glycolysis and tumor growth *via* the miR-185-5p/KLF7 axis (Fig. 8).

Firstly, findings obtained in our study demonstrated that LINC00152 was highly-expressed in PC. This is unsurprising as another study documented robust expressions of LINC00152 in PDAC, which augmented tumor invasion and growth 24. Moreover, LINC00152 is known to exert pernicious effects on a number of malignancies by means of mediating cellular activities, augmenting lymph node metastasis and predicting a high oncology recurrence and unsatisfactory OS 25, 26. In addition, further experimentation in our study revealed that LINC00152 knockdown could inhibit PC cell glycolysis, as evidenced by declined glucose consumption, lactic acid production and ATP levels and decreased levels of HK2, PFKBF3 and PDK1. Glycolysis, a principal transcription factor metabolic course of ATP generation, has also been previously illustrated to promote cancer cell growth, while being activated in multiple human cancers, including PDAC 27. Meanwhile, fluent glucose consumption and a substantial amount of lactic acid production are further known to be precipitated as a result of glycolysis functioning as a major process for fatal carcinoma to meet energy demands 19. On the other hand, activation of HK2, PFKBF3 and PDK1 levels were previously indicated to augment glycolysis 28. Interestingly, a previous report further illustrated that LINC00152 elevated glycolysis in gastric cancer, which is very much in accordance with our findings 29. Although the role of LINC00152 in PC glycolysis remains in the dark, we may confidently draw a conclusion from the above-listed evidences that LINC00152 served as an oncogenic factor in PC by accelerating glycolysis.

Furthermore, another important finding in our study was the ability of LINC00152 to sponge miR-185-5p. Similarly, a previous study indicated that LINC00152 functioned as ceRNA to sponge miR-497 in papillary thyroid carcinoma to encourage tumor development, such that exhaustion of LINC00152 brought about a limiting effect on colony formation and cancer cell expansion 30. In addition, LINC00152 is capable of serving as a tumor driver in hepatocellular carcinoma by sponging miR-193a 31. Together, these evidences suggest that it is highly certain that LINC00152 is implicated in different carcinomas by virtue of serving as an upstream cytokine in the ceRNA network. On the contrary, sponging of miR-185-5p by its target gene is known to exert an augmenting effect on angiogenesis and proliferation, eventually exacerbating atherosclerosis 32. Besides, miR-185-5p was previously indicated to connect to prostate cancer associated transcript 6 and chromobox 2 (both *oncogenes*) *via* the ceRNA mechanism in PDAC 15, further consolidating the conducive role of miR-185-5p in PC. Meanwhile, our findings unveiled that over-expression of miR-185-5p suppressed PC cell glycolysis, as evidenced by degraded glucose consumption, lactic acid production and ATP levels as well as diminished HK2, PFKBF3 and PDK1 levels. Similarly, a prior study demonstrated that over-expressed miR-185-5p enhanced sensitivity to drugs and reduced cell growth in non-small cell lung cancer (NSCLC) 33. Additionally, under-expressed miR-185-5p has been documented in various neoplasms, including prostatic cancer and colon cancer 34, 35, which suggests that miR-185-5p might be a diagnostic biomarker and sanguine gene in cancer mitigation. Moreover, robustly-expressed miR-185-5p is capable of reversing fibrosis and inflammation in kidney injuries induced by high glucose 36. More importantly, a recent study reported that miR‑185 was negatively-associated with HK2, which reversed the suppressive role of miR‑185 in lactic acid release and glucose uptake, eventually unleashing glycolysis in osteosarcoma 37. Likewise, miR-185 can induce apoptosis and quench glycolysis in acute myeloid leukemia *via* the down-regulation of glucose uptake, lactic acid secretion and HK2 expression 38. Collectively, these findings suggest that miR-185-5p confers medical benefits against PC.

Additionally, our findings revealed that LINC00152 exhaustion inhibited KLF7 by sponging miR-185-5p. *An et al*. previously unearthed that KLF7 was activated in exacerbated NSCLC *via* the ceRNA network of LINC00668 and miR-193a 39. Similarly, when KLF7 was quenched by miR-450b-3p, gastric cancer tumor growth was attenuated 40. What's noteworthy is that a prior study demonstrated that KLF7 served as a target of miR-185, and was quenched by miR-185 to restrict NSCLC progression 41. Furthermore, our findings highlighted that silencing LINC00152 quenched PC cell glycolysis by down-regulating the expression of KLF7. Also, in regard to PC, a prior study demonstrated KLF7 was closely-associated with poor OS rates in PC patients 42. Since it served as a risky indicator in clinic consequences of malignancies like ovarian cancer, squamous carcinomas and gastric cancer 43-45, it is quite plausible to suggest that that KLF7 acted as a wicked downstream gene of the ceRNA crosstalk in PC.

Finally, *in vivo* experimentation in our study verified that LINC00152 regulated glycolysis through miR-185-5p/KLF7. We first established subcutaneous tumor models in nude mice, and found that silencing LINC00152 significantly decreased the tumor growth rate and tumor weight, positive rate of Ki67, and glycolysis-related enzymes. On the other hand, over-expression of KLF7 annulled the effects of silencing LINC00152. Ki-67 protein is a well-known tumor proliferation marker, with poor levels of Ki67 reflective of attenuated tumor growth 46. Meanwhile, high glycolysis rates of cancer cells are an important factor for the rapid growth of tumor, and the glycolysis of tumor cells is related to the rapid proliferation of tumor cells 47, 48. Altogether, these findings and evidences suggested that silencing LINC00152 can inhibit tumor formation and glycolysis in pancreatic cancer cells through KLF7, and over-expression of KLF7 may promote the proliferation of PC cells *in vivo* by promoting the glycolysis of PC cells, and thus restore the growth of tumors.

In summary, findings uncovered in our study revealed that silencing of LINC00152 alleviated PC glycolysis by up-regulating miR-185-5p and inactivating KLF7. These discoveries provide a therapeutic implication of silencing LINC00152 for PC treatment. We will further investigate the possible mechanism in PC and its potential therapeutic usage in our future endeavors. Currently, our study still remains a preclinical research and comes with its own set of limitations. For instance, we didn't fully study the mechanism of dysregulated miR-185-5p or KLF7 in PC or glycolysis, and the other genes or pathways downstream of miR-185-5p in PC or glycolysis were not explored. In addition, the experimental findings and practical usage need further validation. Nevertheless, our findings shed a new light on the role of LINC00152, miR-185-5p and KLF7 in PC, and highlight the need for further exploration of LINC00152 and PC.

## Figures and Tables

**Table 1:** Primers sequence of qRT-PCR

	Forward Primer (5'-3')	Reverse Primer (5'-3')
miR-185-5p	GGTGGAGAGAAAGGCAGT	TGCGTGTCGTGGAGTC
LINC00152	AGTTACGGAGGACCCAGCAA	GGGCTGAGTCGTGATTTTCG
KLF7	AGACATGCCTTGAATTGGAACG	GGGGTCTAAGCGACGGAAG
U6	GCTTCGGCAGCACATATACTAAAAT	CGCTTCACGAATTTGCGTGTCAT
β-actin	AGCGAGCATCCCCCAAAGTT	GGGCACGAAGGCTCATC ATT

Note: qRT-PCR, quantitative real-time polymerase chain reaction; miR, microRNA; LINC, long intergenic non-protein coding RNA; Krüppel-like factor 7 (KLF7).
